# Demographics, clinical characteristics, and outcomes among hospitalized heart failure patients across different regions of Egypt

**DOI:** 10.1186/s43044-020-00082-0

**Published:** 2020-08-13

**Authors:** Ahmed Hassanin, Mahmoud Hassanein, Ahmed Bendary, Madiha Abdel Maksoud

**Affiliations:** 1 Westchester Medical Center, New York Medical College, Valhalla, USA; 2 Alexandria University, Alexandria, Egypt; 3 Benha University, Benha, Egypt; 4 Colorado School of Public Health, University of Colorado School of Medicine, Aurora, USA

**Keywords:** Heart failure hospitalization, Epidemiology of heart failure, Global health, Egypt

## Abstract

**Background:**

Regional level data on hospitalized heart failure (HHF) patients in Egypt is scarce. The aim of this study was to compare the demographics, clinical characteristics, and outcomes of HHF patients from four distinct geographical regions of Egypt.

**Results:**

Study participants were part of the European Society of Cardiology Heart Failure Long Term (ESC-HF-LT) Registry, which enrolled patients from April 2011 to February 2014. A total of 1661 HHF patients from Egypt were enrolled, of whom 1645 were eligible for analysis: 914 from Alexandria, 249 from Cairo, 409 from the Delta region, and 73 from Upper Egypt.

The mean age ranged from 52.2 to 62.8 years and differed significantly between the 4 groups (*P* < 0.01). Females represented one-third of the cohort (*P* = 0.5 between groups). The prevalence of obesity, diabetes, and hypertension also varied significantly across the groups (*P* < 0.01). The most common etiology of heart failure (HF) was ischemic heart disease. HF with reduced systolic function was the leading type of HF in the 4 groups (*P* = 0.6). The most common valvular abnormality in all regions was mitral regurgitation. For patients with prior history of HF, community-acquired infection was the most common reason for a HF exacerbation in all 4 groups.

In-hospital mortality ranged from 2.9 to 7.7% in the 4 groups (*P* = 0.06). Only Alexandria and Delta groups provided reliable 1-year follow-up data, given low patient retention in Cairo and Upper Egypt groups. At one-year, 32% of patients from Alexandria compared to 22.6% from Delta were re-hospitalized for HF (*P* < 0.01). Mortality at 1 year was also significantly higher in Alexandria compared to Delta, 31.8 vs 13.2% respectively (*P* < 0.01).

**Conclusions:**

HHF patients from different geographic regions of Egypt differed significantly in their demographics, clinical characteristics, and outcomes. Those differences underscore the importance of region-specific HF prevention and management strategies.

## Background

In Egypt, cardiovascular disease (CVD) has been the leading cause of premature death since the 1990s [1]. In 2017, CVD accounted for 46.2% of the overall mortality in Egypt [2]. Given the aging of the population and the success in prolonging the survival of those with coronary events, heart failure (HF) poses an important and growing public health burden.

The European Society of Cardiology Heart Failure Long-Term (ESC-HF-LT) Registry, in which Egypt participated as a member country, provided the first representative data on HF patients in the country [3]. Studies comparing the cohort of heart failure (HF) patients in Egypt to the European cohorts, as well as gender differences of HF patients within Egypt, have been published [4, 5]. However, no study to date has compared the characteristics and outcomes of HF patients across the different regions of Egypt. Regional level data is particularly important in a populous country like Egypt, which has a population of over 100 million with large disparities in socioeconomic and geographical factors.

The aim of this study is to compare the demographics, clinical characteristics, treatment patterns, and outcomes of patients hospitalized for heart failure (HHF), for patients from 4 distinct geographical regions of Egypt: Alexandria, Greater Cairo, Delta governorates, and Upper Egypt.

## Methods

### Registry population

The ESC-HF-LT Registry has been described elsewhere [3]. Briefly, this was a prospective, multi-center, observational study of patients presenting to cardiology centers across several European and Mediterranean countries. Twenty centers, representing diverse geographic regions of Egypt (Mediterranean coast, Nile Delta, Greater Cairo, Upper Egypt, and Suez Canal regions) participated in the registry. Site selection was aimed to target a sample of hospitals of different levels of complexity that was representative of Egyptian reality. Nine participating centers were university hospitals; 7 centers had neither catheterization laboratories nor cardiac surgery facilities.

Between April 2011 and February 2014, a total of 1661 HHF patients from Egypt were enrolled in the ESC-HF-LT Registry. HF was diagnosed according to the clinical judgment of participating centers’ responsible cardiologist. Study patients were required to be 18 years and older in order to consent to the study. The Registry was approved by each local Institutional Review Board according to the rules of each participating center.

### Study population

**Fig. 1:**
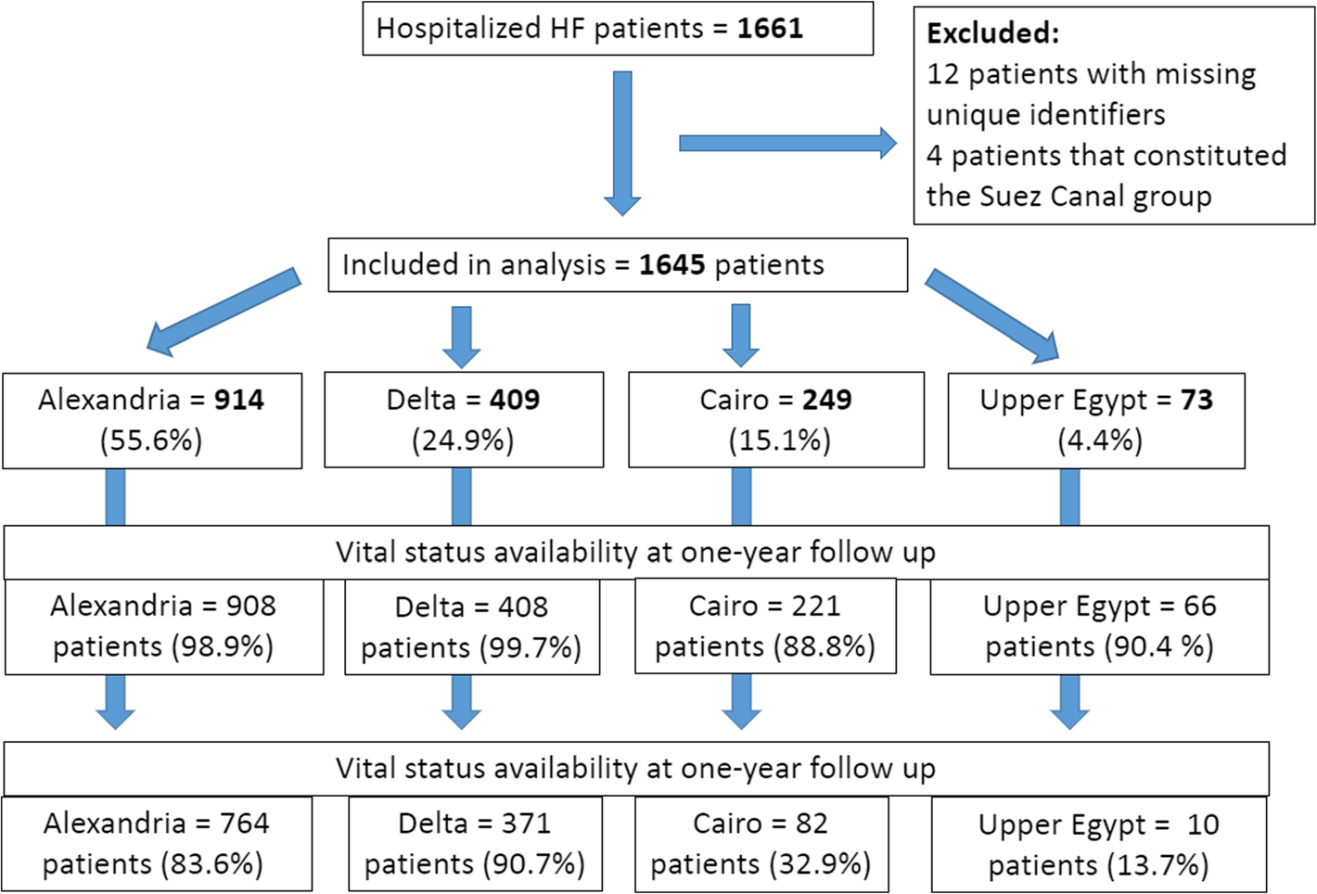
Study flow chart

**Fig. 2:**
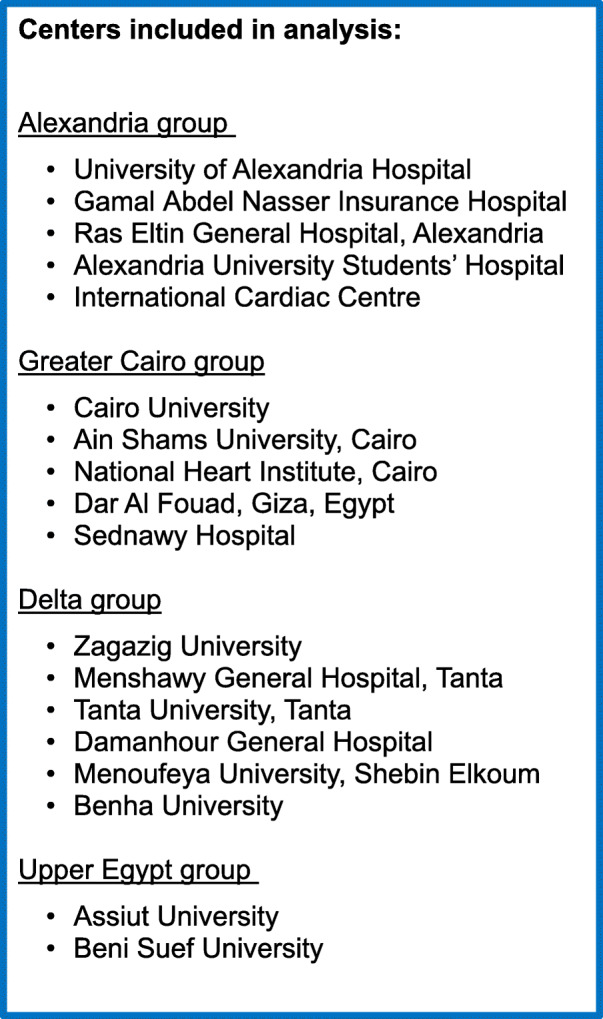
Enrolling centers

Of the 1661 HHF patients from Egypt, 12 patients were excluded for missing unique identifiers, and 4 patients representing the HHF from the Suez Canal region were also excluded as the group was too small to be representative of the region. In total, 1645 patients were eligible for analysis: 914 from Alexandria (5 centers), 249 from Cairo (5 centers), 409 from the Delta region (6 centers), and 73 from Upper Egypt (2 center). See Fig. 1 for the study flow diagram and Fig. 2 for the list of the enrolling centers.

### Statistical analysis

Continuous variables that followed a normal distribution were reported as mean ± standard deviation (SD), while continuous variables following a non-normal distribution were presented as median ± interquartile range (IQR). Categorical variables were reported as percentages. Kruskal–Wallis test was used to compare continuous variables between groups. Chi-square was used to compare categorical variables between groups. A *P* value of < 0.05 was considered to represent statistical significance. All tests were two-sided. Analyses were performed using SAS version 9.4 (SAS Institute Inc., Cary, NC, USA).

## Results

### Demographics, cardiovascular risk factors, and comorbidities (Table 1)

The mean age of HHF patients at enrollment varied significantly between the 4 groups, with the highest age being in Alexandria, 62.3 (± 11.6) years, and the lowest in Upper Egypt, 51.2 (± 11.2) years (*P* < 0.01). Females represented one-third of the entire cohort, and that did not differ significantly across the 4 regions (*P* = 0.5).

**Table 1:** Demographics, cardiovascular risk factors, and comorbidities

	Total population	Alexandria	Cairo	Delta	Upper Egypt	***P*** value
**Number of patients**	1645	914	249	409	73	
Demographics and traditional cardiovascular risk factors						
**Age, mean (± SD)**	60.1 (± 12.1)	62.3 (± 11.6)	56.2 (± 13.2)	59.9 (± 12.1)	51.2 (± 11.2)	< 0.0001
**Age ≥ 70, %**	21.1	25.1	12.5	20.5	4.1	< 0.0001
**Females %**	32.0	31.8	34.9	31.5	26.1	0.56
**BMI (kg/m**^**2**^**), median (IQR)**	29.4 (26.5–33.2)	29.7 (26.6–34.2)	29.2 (26.1–33.20)	29.4 (27.1–32.3)	26.1 (24.3–29.1)	< 0.0001
**BMI ≥ 30, %**	45.5	48.7	40.2	46.2	19.2	< 0.0001
**Smoker (current/ever), %**	59.3	63.0	53.2	55.8	52.8	0.01
**Female smokers, %**	8.3	8.83	0.4	0.0	0.19	< 0.0001
**Diabetes**	44.2	48.6	45.6	35.5	34.3	< 0.0001
**Insulin-dependent diabetes (as % of all patients)**	21.5	18.9	30.6	22.3	19.2	< 0.0001
**Hypertension, %**	42.1	51.8	26.2	34.5	17.8	< 0.0001
Comorbidities						
**MI/angina, %**	65.9	74.3	52.8	59.9	38.4	< 0.0001
**History of PCI, %**	10.0	10.1	19.0	5.4	7.6	< 0.0001
**History of CABG, %**	4.3	4.2	7.7	2.7	4.6	< 0.0001
**Atrial fibrillation, %**	25.1	22.4	29.5	27.9	28.8	0.04
**PAD. %**	5.1	6.5	4.4	2.2	6.9	0.01
**Stroke/TIA, %**	7.5	8.1	8.9	4.7	11.0	0.06
**Chronic kidney disease, %**	16.6	21.1	17.7	7.8	6.9	< 0.0001
**COPD, %**	14.1	15.6	15.2	12.2	1.37	0.005
**Hepatic dysfunction, %**	9.1	10.7	8.06	5.6	12.3	0.02
**Chronic hepatitis C, %**	8.5	10.1	8.8	5.6	4.6	0.2
**Hemoglobin (g/dL) median, IQR**	11.8 (10.3–13)	11.9 (10.3–13)	12.0 (10.5–13.2)	11.7 (10.5–13)	11.0 (10–12)	0.02
**Hemoglobin ≤ 12 g/dL, %**	58.8	57.5	53.3	61.1	79.4	0.001

*SD* standard deviation, *BMI* body mass index, *MI* myocardial infarction, *PCI* percutaneous coronary intervention, *CABG* coronary artery bypass surgery, *PAD* peripheral arterial disease, *TIA* transient ischemic attack, *COPD* chronic obstructive pulmonary disease, *IQR* interquartile range

Cardiometabolic risk factor prevalence varied significantly between HHF patients across groups. The prevalence of obesity, defined as a body mass index (BMI) ≥ 30 kg/m^2^, ranged from 40 to 49% in Alexandra, Cairo, and the Delta but was much less common in Upper Egypt, 19%, (*P* < 0.0001). Diabetes prevalence was > 45% in Alexandria and Cairo and 35% in the Delta and Upper Egypt (*P* < 0.01). The prevalence of hypertension was highest in Alexandria, 51.8%, and lowest in UE, 17.8% (*P* < 0.01). Smoking (current/ever) was highly prevalent, > 50% in all regions, but uncommon among females in the cohort.

The prevalence of atrial fibrillation was similar between the groups and ranged from 22.4 to 29.5% (*P* = 0.04). The prevalence of chronic kidney disease, defined as serum creatinine > 1.5 mg/dL, was highest in Alexandria and Cairo, and much lower in Delta and Upper Egypt (*P* < 0.0001). Anemia, defined as hemoglobin ≤ 12, was very common in Upper Egypt—seen in nearly 80% of the patients in this group.

### Etiology and HF precipitating factors (Table 2, Fig. 3)

The most common etiology of HF overall was ischemic heart disease; however, the prevalence differed significantly ranging from 72.5% in Alexandria to 40.9% in Upper Egypt (*P* < 0.01). The second most common etiology in Alexandria and Delta was dilated cardiomyopathy (DCM). In Cairo, DCM and valvular heart disease came in the second position, whereas in Upper Egypt, valvular heart disease was the second most common etiology accounting for a quarter of the cases.

**Table 2:** Etiology and heart failure precipitating factors

	Total population	Alexandria	Cairo	Delta	Upper Egypt	***P*** value
**Number of patients**	1645	914	249	409	73	
Etiology, %						
**Ischemic**	65.5	72.5	53.4	61.4	40.9	< 0.0001
**Dilated**	16.8	11.2	16.2	28.4	23.9	< 0.0001
**Valvular**	8.7	6.2	18.2	5.9	25.4	< 0.0001
**Hypertensive**	3.6	4.9	2.4	1.0	5.6	0.002
**Other**	5.4	5.2	9.8	3.3	4.2	0.004
HF precipitating factors for established cases, %						
**ACS**	14.0	18.6	8.1	9.3	6.0	< 0.0001
**Infection**	34.3	35.2	24.3	38.4	38.4	0.02
**Uncontrolled hypertension**	17.2	25.1	6.9	8.9	4.0	< 0.0001
**Anemia**	25.5	35.7	13.9	12.7	14.0	< 0.0001
**Renal dysfunction**	14.9	17.7	17.3	8.4	6.0	0.002
**Atrial fibrillation**	19.8	13.7	23.7	31.2	20.0	< 0.0001
**Non-adherence with medication**	10.4	4.0	12.8	20.7	24.5	< 0.0001

*ACS* acute coronary syndrome

**Fig. 3:**
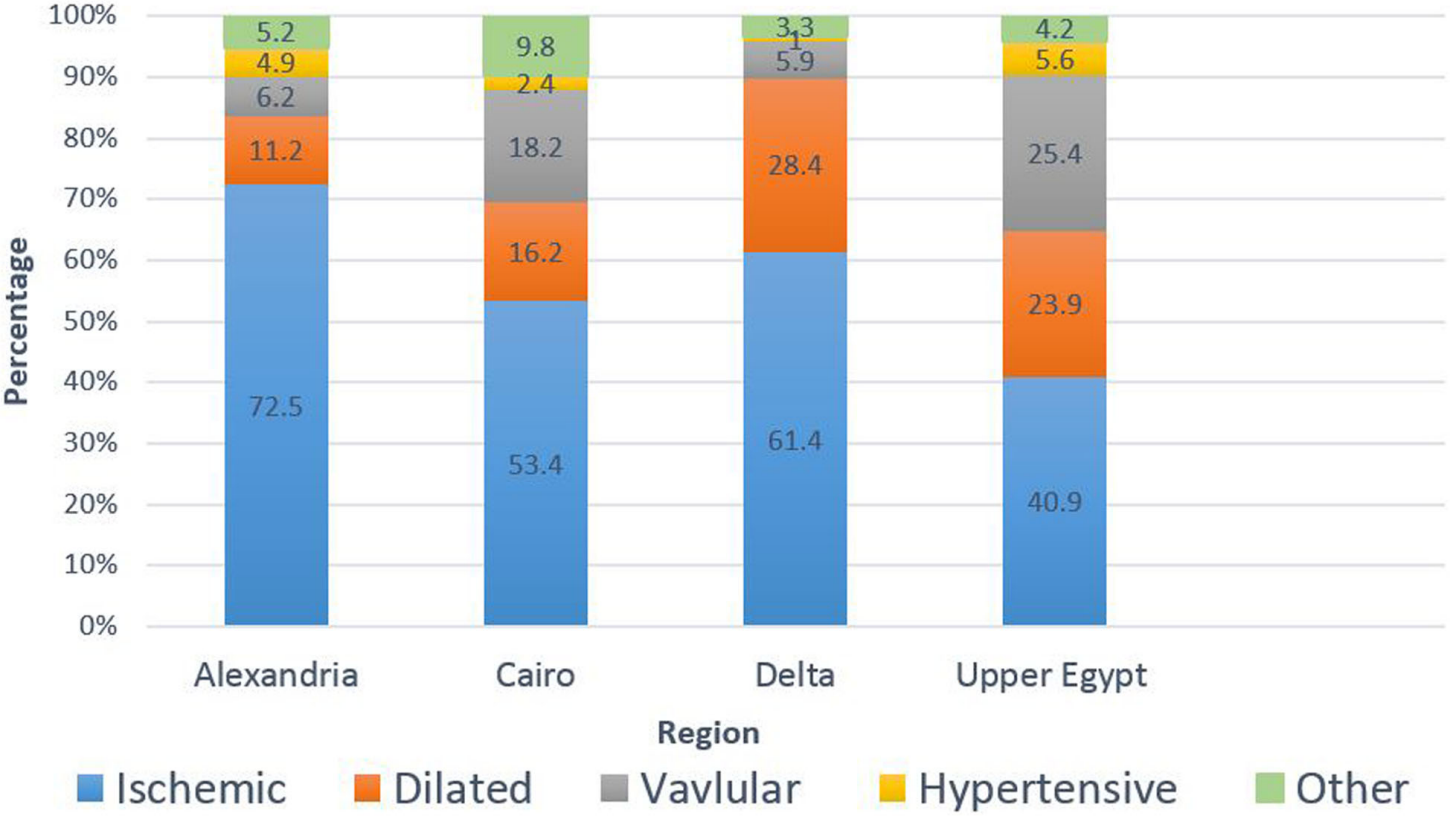
Etiology of heart failure

In HHF patients with established HF prior to enrollment, community-acquired infections were considered to be the leading cause for HF decompensation in all 4 groups. In Alexandria, anemia was found to be equally important cause for decompensation. The second most cause for decompensation was atrial fibrillation in Cairo and Delta and medication non-adherence in Upper Egypt.

### Clinical presentation (Table 3)

The majority of the patients, 85%, were admitted after being sent directly to the hospital from the outpatient cardiology or general practitioners’ office. Only 11.6% of patients utilized an ambulance for transportation to the hospital, while the vast majority used another means of transportation. The majority (51%) of patients were admitted directly to the cardiac intensive care unit, while the rest were admitted to the cardiac ward. The mode of transportation, level of care on admission, and the referral type did vary significantly between the geographical groups studied.

**Table 3:** Clinical presentation

	Total population	Alexandria	Cairo	Delta	Upper Egypt	***P*** value
**Number of patients**	1645	914	249	409	73	
**HF: new onset, %**	38.0	39.2	29.5	42.1	28.6	0.004
**HF: worsening, %**	62.0	60.8	70.5	58.0	71.4	0.004
**NYHA functional class III or IV**	92.8	91.4	90.8	96.6	97.2	0.002
**HR, median, IQR**	100 (90–112)	104 (94–116)	90 (80–105)	100 (90–110)	100 (80–110)	< 0.0001
**SBP median, IQR**	130 (110–150)	140 (120–164)	115 (110–130)	120 (100–140)	110 (100–130)	< 0.0001
**Sodium, mean (± SD)**	137.1 (± 6.3)​	138.3 (± 5.9)	134.6 (± 6.2)	136.2 (± 5.8)	132.9 (± 7.4)	< 0.001
Clinical presentation, %						
**Cardiogenic shock/HF**	3.7	2.3	4.1	6.1	7.0	< 0.0001
**ACS/HF**	19.6	26.6	9.8	12.2	5.6	< 0.0001
**Decompensated HF**	54.8	47.1	68.2	61.1	71.8	0.15
**Hypertensive HF**	4.7	5.6	4.1	3.4	1.4	0.16
**Pulmonary edema/HF**	12.7	12.4	9.4	15.2	14.1	
**Right-sided HF**	4.5	6.0	4.5	2.0	0	0.005

*HF* heart failure, *NYHA* New York Heart Association, *HR* heart rate, *SBP* systolic blood pressure, *SD* standard deviation

Approximately two-thirds of patients across the four groups had previously diagnosed HF. The most common phenotypic presentation in all 4 groups was decompensated HF. In the Alexandria and Cairo groups, concomitant acute coronary syndrome (ACS) and HF was the second most common phenotype of presentation. Whereas in the Delta and Upper Egypt, pulmonary edema was the second most common phenotype of presentation.

### Workup and management (Tables 4 and 5)

Echocardiographic assessment was performed in the majority of patients on admission across all 4 regions. The mean left ventricular ejection fraction was 36%, which did not differ significantly between groups (*P* = 0.06). More than half of the patients in all 4 groups had HF with reduced ejection fraction (HFrEF). The most prevalent valvular abnormality in all four groups was mitral regurgitation. Aortic stenosis was seen in 10% of patients from Upper Egypt. Mitral stenosis (MS) prevalence was not captured in this Registry as MS is rare across ESC member countries—except for Egypt.

**Table 4:** Workup

	Total population	Alexandria	Cairo	Delta	Upper Egypt	***P*** value
**Number of patients**	1645	914	249	409	73	
Echocardiographic findings						
**Echo performed on admission, %**	76.5	73.2	65.0	90.2	74.6	< 0.0001
**LVEF %, on admission, mean (±SD)**	38.6 (± 12.8)	38.8 (± 12.7)	38 (± 16.0)	37.9 (± 10.3)	39.6 (± 11.3)	0.6
**HFpEF, % (of the total cohort)**	23.5	18.2	28.7	12.2	21.6	< 0.0001
**HFmrEF, % (of the total cohort)**	19.2	24.8	10.9	30.6	27.5	0.007
**HFrEF, % (of the total cohort)**	57.3	57	60.4	57.2	51	0.54
**MR moderate/severe, %**	61.5	54.3	46.7	78.5	72.0	< 0.0001
**AS moderate/severe**	3.3	3.2	3.7	2.5	10.0	< 0.0001
**AR moderate/severe, %**	5.6	3.2	4.4	10.1	8.0	< 0.0001
**TR moderate/severe, %**	38.7	32.2	38.4	48.1	56.0	< 0.0001
Other diagnostic tools						
**RHC, %**	0.3	0.4	0.00	0.00	0.00	0.4
**Coronary angiography, %**	7.7	7.3	15.0	5.6	3.0	< 0.0001

*LVEF* left ventricular ejection fraction, *HFpEF* heart failure with preserved ejection fraction, *HFmrEF* heart failure with mid-range ejection fraction, *HFrEF* heart failure with reduced ejection fraction, *MR* mitral regurgitation, *AS* aortic stenosis, *AR* aortic regurgitation, *TR* tricuspid regurgitation, *RHC* right heart catheterization

**Table 5:** Management

	Total population	Alexandria	Cairo	Delta	Upper Egypt	***P*** value
**Number of patients**	1645	914	249	409	73	
**ACEi, %**	76.4	79.6	57.4	79.2	79.3	< 0.0001
**ARB, %**	10.1	8.5	15.5	11.5	3.5	0.1
**ACEi/ARB, %**	86.5	88.1	72.9	90.7	82.2	< 0.0001
**BB, %**	64.0	70.1	53.7	55.4	71.4	< 0.0001
**MRA, % (if indicated)**	78.7	71.4	82.4	88.0	90.0	0.0002
**Diuretics, %**	76.6	71.2	78.0	86.0	85.7	< 0.0001
**CCB, %**	8.3	10.5	6.1	4.7	8.8	0.003
**Nitrites, %**	50.5	57.9	34.9	48.0	12.3	< 0.0001
**Digitalis, %**	36.1	25.5	47.9	52.2	38.6	< 0.0001
**Amiodarone, %**	11.3	8.3	13.0	15.8	11.3	0.0002
**Ivabradine, %**	7.1	3.0	10.7	12.8	15.8	< 0.0001
**Statins, %**	70.5	80.6	50.5	65.0	31.6	< 0.0001
**Anti-platlets, %**	78.5	85.6	65.4	74.5	49.1	< 0.0001
**Anti-coagulants, %**	32.7	23.7	39.5	49.0	29.8	< 0.0001
Cardiac devices utilization						
**CRT indicated, %**	14.0	17.5	8.2	11.3	1.5	< 0.0001
**CRT planned, %**	2.5	1.0	2.7	3.7	1.5	0.08
**ICD indicated, %**	6.6	5.2	9.1	9.3	0.0	< 0.0001
**ICD planned %**	1.5	0.1	2.4	2.4	0.0	0.65

*ACEi* angiotensin-converting enzyme inhibitors, *ARB* angiotensin receptor blocker, *MRA* mineralocorticoid receptor antagonist, *CRT* cardiac resynchronization therapy, *ICD* intracardiac defibrillator

The utilization of right cardiac catheterization was very rare, < 1%, across regions, whereas coronary angiography was utilized in a small percentage of patients, ranging from 15% in Cairo to 3% in Upper Egypt.

The utilization of angiotensin-converting enzyme inhibitors (ACEi) or aldosterone receptor blockers (ARB) was generally high. The utilization of beta-blockers was higher in Alexandria and Upper Egypt as compared to Cairo and Delta regions (*P* < 0.0001). In patients with a left ventricular ejection fraction < 35%, utilization of mineralocorticoid was generally high across the four regions. Digoxin use differed across the four regions; it was highest in Delta, 52%, and lowest in Alexandria, 25% (*P* < 0.0001). Statins use was generally low except in Alexandria.

Cardiac resynchronization therapy (CRT) or implantable cardioverter-defibrillator (ICD) devices were implanted in only 11 patients of the entire cohort. In patients in which device therapy was indicated, only a very small fraction of patients across the 4 regions were planned for the device implantation.

### Outcomes (Table 6)

The median length of stay was 5 days in Alexandria, 7 days in Cairo, and 6 days in Delta and Upper Egypt (*P* < 0.0001). In-hospital mortality was 2.9, 5.2, 6.1, and 7.7% for Delta, Alexandria, Upper Egypt, and Cairo groups, respectively (*P* = 0.06). Due to the high proportion of patients lost to follow-up in Cairo and Upper Egypt, only Alexandria and Delta groups provided reliable 1-year follow-up data (Fig. 1). At one-year, 32% of patients from Alexandria compared to 22.6% from Delta were rehospitalized for HF (*P* < 0.01). Mortality at 1-year was also significantly higher in Alexandria compared to Delta, 31.8 vs 13.2%, respectively (*P* < 0.01).

**Table 6:** Outcomes

	Total population	Alexandria	Cairo	Delta	Upper Egypt	***P*** value
**Number of patients**	1645	914	249	409	73	
**Length of hospital Stay median, IQR**	5 (4–7)	5 (4–7)	7 (5–9)	6 (4–7)	6 (5–9)	< 0.0001
**In-hospital mortality, %**	5.00	5.2	7.7	2.9	6.1	0.06
**Patients hospitalized at 1-year F/U, %**	29.8	32.0	42.2^a^	22.6	20.0^b^	< 0.0001
**Mortality at 1-year F/U, %**	26.6	31.8	34.12^a^	13.2	60.0^b^	< 0.0001

*IQR* interquartile range, *F/U* follow-up, *HF* heart failure

^a^Only 32.9% of patients were successfully contacted at 1-year follow-up

^b^Only 13.7% of the patients were successfully contacted at 1-year follow-up

## Discussion

The main findings of this study are the following:

- The mean age, prevalence of cardiometabolic risk factors, and medical comorbidities differed significantly across regions.
- Ischemic heart disease was the leading etiology, and HFrEF was the most common type of HF, across the 4 regions.
- The rate of prescription of guideline-directed medical therapy was high on discharge across regions. However, cardiac device utilization was uncommon.
- In-hospital mortality did not statistically differ between regions. However, 1-year mortality and rates of re-hospitalization differed significantly.

To our knowledge, this is the first analysis of HHF patients in Egypt to compare the demographics, clinical presentation, primary etiology, management, and outcomes across different regions of the country. The diversity of enrolling hospitals (university, non-university, and community) reflects the actual practice and management of HHF in those regions.

Many low- and middle-income countries suffer from uneven distribution of wealth and resources. In 2015, 27.8% of Egyptians lived under the national poverty line of $1.5 daily, and over 50% of those who live in rural Upper Egypt are under the poverty line [6]. It is not surprising that this wealth gap would be reflected in the demographic and clinical features of HHF patients across the different regions of Egypt.

The mean age of the Egyptian cohort was 10-year youngers than that of the rest of the ESC-HF-LT Registry regions, but similar to that of patients in the Saudi HF registry [7]. In the Egyptian cross-sectional CardioRisk project, premature ACS was highly prevalent in Egyptians, seen among 46% of men aged less than 55 years and in 67% of women aged < 65 years [8], and this could be considered a potential contributing factor to the earlier presentation for HF seen in the Egyptian cohort. Within our study’s cohort, the mean age varied by almost 10 years between regions, with the highest age in Alexandria, 62.3 (± 11.6) years, and the lowest in Upper Egypt, 51.2 (± 11.2) years (*P* < 0.01).

Traditional cardiometabolic risk factors were highly prevalent across all groups. Of particular concern is tobacco use, which was ubiquitous among males across the 4 regions. In the Egyptian cross-sectional CardioRisk project [8], 62% of men and 5% of women presenting with ACS were current smokers. Exposure to smoking in Egypt is high across all wealth categories as reported in the Egyptian Health Issues Survey in 2015 [9]. Diabetes was particularly concentrated in the more urban regions of Alexandria and Cairo. Obesity was also highly prevalent except in Upper Egypt. A previous publication from this cohort has shown that women had a higher BMI than men (32.5 ± 9.0 vs 29.3 ± 4.9, *P* < 0.001), and 66% of Egyptian women with HF were obese [4]. Hypertension was seen in half of the patients in the Alexandria region, and this may be related to dietary patterns for the region or the higher mean age in this group.

Anemia was highly prevalent across regions, especially in Upper Egypt where it was seen in almost 80% of patients. Hassanein et al. have reported that anemia is particularly prevalent among Egyptian women with HF as compared to men, 35.4% versus 18.9%, respectively [4]. In a systematic review and meta-analysis by Groenveld et al. [10], anemia was associated with an increased risk of mortality in both systolic and diastolic HF. Hepatic dysfunction and chronic hepatitis C were common across all regions.

Ischemic cardiomyopathy was the leading etiology for HF in all 4 regions. Valvular heart disease was responsible for 25% of cases of HF in Upper Egypt. Although rheumatic heart disease (RHD) prevalence was not captured in this registry, we suspect that it is the main cause for valvular heart disease seen in Upper Egypt given the high prevalence of aortic stenosis seen in that region’s patients despite their young age. Previous echocardiographic screening studies from Upper Egypt have suggested that the prevalence of RHD is 31 cases per 1000 children [11].

Patients from Upper Egypt had several demographic and clinical characteristics that distinguished them from the other three regions. They were generally younger, had lower BMI, higher prevalence of anemia, lower prevalence of diabetes, and more valvular heart disease. We suspect that those characteristics are in part due to poor access to health care and limited financial resources the is ubiquitous in Egypt’s rural regions. The younger age of onset for HF in Upper Egypt is possibly related to the limited access to coronary artery disease primary and secondary prevention strategies, and the high prevalence of valvular heart disease in the region. The lower BMI and high prevalence of anemia are possibly attributable to malnutrition. The low prevalence of diabetes is likely related to the low BMI and maybe confounded by the younger age patients enrolled in this region.

Prescription of guideline-directed HF medical therapy was generally high in all regions as compared to other countries in the ESC-HF-LT Registry. Beta-blockers use was an exception and was particularly underutilized in the Cairo and Delta regions. Digoxin appears to be overprescribed: its use was seen in 36% of the entire Egyptian cohort as compared to just 21% of the other countries in ESC-HF-LT Registry [12]. The utilization of cardiac devices was generally very rare. In cases were CRT and ICDs were indicated, only 25% of patients were planning on having the procedure with the main obstacle stated being cost.

The median length of hospital stay across the entire cohort was relatively short at 5 days, ranging from 5 to 7 days. This is shorter than the median length of stay of 7 days in other countries participating in the ESC-HF-LT Registry [13], perhaps reflecting the scarcity of beds and the need for rapid discharges. Egypt has 1.6 hospital beds per 1000 people as compared to 5.6 beds per 1000 people in the European Union [14]. One in 3 patients in Alexandria and one in 5 patients in the Delta were rehospitalized within 1 year of enrollment. One-year mortality is Alexandria was 31.8% as compared to just 12.3% in Delta. This likely represents referral bias whereas more complicated cases are referred to urban centers. Although the 1-year mortality in Alexandria was similar to the other regions participating in the ESC-HF-LT Registry [13], the patient from Alexandria was 10 years younger, indicating higher risk-adjusted mortality in the Egyptian cohort.

There are several limitations of this registry. First, the diagnosis of HF was made by each center’s practicing physician and was not validated centrally. Only 24.3% of HHF patients with presumed ischemic HF cases were verified angiographically. Second, patients enrolled in the registry did not include HHF patients admitted to other non-cardiac wards in the hospital. Third, the relatively small number of patients enrolled from Upper Egypt may not be adequately representative of the region. Forth, 1-year follow-up data was scarce in the region of Cairo and Upper Egypt. Finally, the Suez Canal region and frontier regions, such as Sinai, were not represented in this analysis.

## Conclusion

In conclusion, HHF patients throughout Egypt differ in their demographic and clinical characteristics, as well as their outcome depending on the region. This highlights the importance of region-specific prevention and management strategies.

## Data Availability

The datasets generated and/or analyzed during the current study are available on request from the European Society of Cardiology/EURObservational Research Programme.

## Abbreviations

ESC-HF-LT: European Society of Cardiology Heart Failure Long-Term
HHF: Hospitalized heart failure
HF: Heart failure
CVD: Cardiovascular disease
SD: Standard deviation
IQR: Interquartile range
BMI: Body mass index
DCM: Dilated cardiomyopathy
ACS: Acute coronary syndrome
HFrEF: Heart failure with reduced ejection fraction
MS: Mitral stenosis
RHD: Rheumatic heart disease
ACEi: Angiotensin-converting enzyme inhibitors
ARB: Aldosterone receptor blockers
CRT: Cardiac resynchronization therapy
ICD: Implantable cardioverter-defibrillator
